# Spiking Neural Network for Augmenting Electroencephalographic Data for Brain Computer Interfaces

**DOI:** 10.3389/fnins.2021.651762

**Published:** 2021-04-01

**Authors:** Sai Kalyan Ranga Singanamalla, Chin-Teng Lin

**Affiliations:** ^1^Computational Intelligence and Brain Computer Interface Lab, School of Computer Science, University of Technology Sydney, Sydney, NSW, Australia; ^2^Centre for Artificial Intelligence, University of Technology Sydney, Sydney, NSW, Australia

**Keywords:** spiking neural network, electroencephalography, brain computer interface, motor imagery, data augmentation

## Abstract

With the advent of advanced machine learning methods, the performance of brain–computer interfaces (BCIs) has improved unprecedentedly. However, electroencephalography (EEG), a commonly used brain imaging method for BCI, is characterized by a tedious experimental setup, frequent data loss due to artifacts, and is time consuming for bulk trial recordings to take advantage of the capabilities of deep learning classifiers. Some studies have tried to address this issue by generating artificial EEG signals. However, a few of these methods are limited in retaining the prominent features or biomarker of the signal. And, other deep learning-based generative methods require a huge number of samples for training, and a majority of these models can handle data augmentation of one category or class of data at any training session. Therefore, there exists a necessity for a generative model that can generate synthetic EEG samples with as few available trials as possible and generate multi-class while retaining the biomarker of the signal. Since EEG signal represents an accumulation of action potentials from neuronal populations beneath the scalp surface and as spiking neural network (SNN), a biologically closer artificial neural network, communicates via spiking behavior, we propose an SNN-based approach using surrogate-gradient descent learning to reconstruct and generate multi-class artificial EEG signals from just a few original samples. The network was employed for augmenting motor imagery (MI) and steady-state visually evoked potential (SSVEP) data. These artificial data are further validated through classification and correlation metrics to assess its resemblance with original data and in-turn enhanced the MI classification performance.

## 1. Introduction

Brain–computer interfaces (BCIs) are a form of human–computer interaction through which users can communicate with an external device or application, such as wheelchair navigation, playing games, operating prosthetics, or using a keyboard speller, via their thoughts (Donoghue, 2002; Moore, 2003; Schalk et al., 2004). The fundamental principle of a BCI system is interpreting brain signals to extract reliable markers for decoding the user intentions and translate this information as a command to an external application. In BCI applications, electroencephalography (EEG) is the most commonly used brain imaging method for monitoring brain signals due to its portability and high temporal resolution (Cincotti et al., 2008; Alwasiti et al., 2010; Fazli et al., 2012).

EEG–BCI systems can employ different modalities, such as motor imagery (MI) (Marchesotti et al., 2016), steady-state visually evoked potential (SSVEP) (Nakanishi et al., 2014), and P300 potentials (Jin et al., 2015). The recent developments in deep learning have resulted in an unprecedented improvement in the performance of BCI systems (Zhang et al., 2017; Chiarelli et al., 2018; Schwemmer et al., 2018). However, such machine learning techniques require abundant user-specific labeled data samples for effective training, and recording a large sample size is experimentally a tedious process. Additionally, EEG data are often contaminated with noise and artifacts, leading to the exclusion of many samples during the data pre-processing steps. Generating synthetic EEG samples could aid in the training of machine learning algorithms. Lotte (2011) generated synthetic EEG trials by chunking each original EEG trial into a different segment and then combine the segments from different trials to form a new artificial sample. But this process results in a trade-off with the loss of spectral-domain features in the artificial signal and requires a careful division of segments to maintain the temporal features. In another study, Dinarès-Ferran et al. (2018) employed empirical mode decomposition (EMD), in which a signal is decomposed into a finite number of intrinsic function, a nonlinear oscillatory signal. These decomposed signals from different EEG trials are again composed to produce a new EEG sample. Though this method aims to retain the spectral domain features, the study stated this method could often produce irrelevant artificial samples.

In the recent decade, various deep learning-based generative models were formulated especially for image and speech synthesis. The most prominent of these models include generative adversarial networks (GAN) and its variants, variational autoencoders (VAE), etc. Such models have been explored for generating artificial EEG data with a focus on performance enhancement in BCI applications. For example, Aznan et al. (2019) generated artificial SSVEP data using deep convolution GAN (DCGAN), Wassertian GAN (W-GAN), and VAE. Similarly, Hartmann et al. (2018) used modified W-GANs to stabilize its training process in generated left-hand motor imagery data. However, these generative models can be trained to produce only one class of data. Although GAN-based networks have shown promising output, training the model is complex due to GAN instability, fine-tuning of hyperparameters, and a sufficiently large number of samples are needed for training. And, we believe requiring huge data for generating even more samples is counterintuitive. Therefore, a new generative model is required that can produce multi-class artificial EEG data with as few available original data samples as possible.

Humans process information through ensembles of spiking neurons and the reorganization of collective chaotic neuronal activity to produce varied behaviors and actions remain a key neuroscience research topic (Sussillo and Abbott, 2009; Churchland et al., 2012; Gilra and Gerstner, 2017; Nicola and Clopath, 2017). Nicola and Clopath (2017) enforced such behavior in a recurrent spiking neural network (SNN) using FORCE, a supervised learning method, and reproduced an array of signals such as sinusoidal waves, Lorenz attractor, Ode to Joy, bird song, and movie replay mimicking the hippocampal region. In another study, Ingrosso and Abbott (2019) focused on dynamically balanced recurrent spiking networks for reconstructing motion tracking signals using bounded constrained coordinate descent optimization.

Inspired by these studies, understanding the underlying spiking dynamics in EEG generation could potentially advance BCI systems. Previously adopted methods for signal reconstruction, such as recursive least square (RLS), have been shown to train SNNs with remarkable performance. However, these methods are usually suitable for periodic signals and require finely tuned hyperparameters for training different signals, i.e., the same set of hyperparameters cannot account for training different signals. In contrast, EEG is non-stationary and has high variance. To address these issues, we implemented a feedforward SNN and trained the network via surrogate-gradient descent (Zenke and Ganguli, 2018; Neftci et al., 2019) method to reconstruct EEG template and, in turn, generate synthetic EEG signals.

The non-stationarity of EEG signals and their uniqueness across recorded trials for a given stimulus pose a challenge in reconstructing the signal and in generating a synthetic dataset in which each sample is different. The famous rodent spike train repeatability experiment (Mainen and Sejnowski, 1995) has shown that the same stimulus can elicit different spike trains (with slight changes in spike timings) at neocortical neurons owing to the variability either with unknown information flow from other circuits or intrinsic background noise in the system. Inspired by this concept, we aimed to influence the trained SNN model through an external neural perturbation layer as background noise. By modulating the perturbation layer's noise level, the proposed model was able to (theoretically) generate an unlimited number of samples of MI and SSVEP. One of the main use of synthetic data in machine learning is to improve classifier performance. The synthetic MI-EEG data enhanced the classification accuracy when used for training the classifier. The major contributions of this work are threefold: (1) the development of an SNN that is agnostic to the EEG signal modality, i.e., the same model architecture to accommodate varied EEG signals (e.g., MI and SSVEP) without extensively change the hyperparameters, (2) the generation of multi-class synthetic motor imagery EEG data from only a few original samples, and (3) validating the artificial MI data through classification and performance enhancement.

## 2. Methods

**Figure 1:**
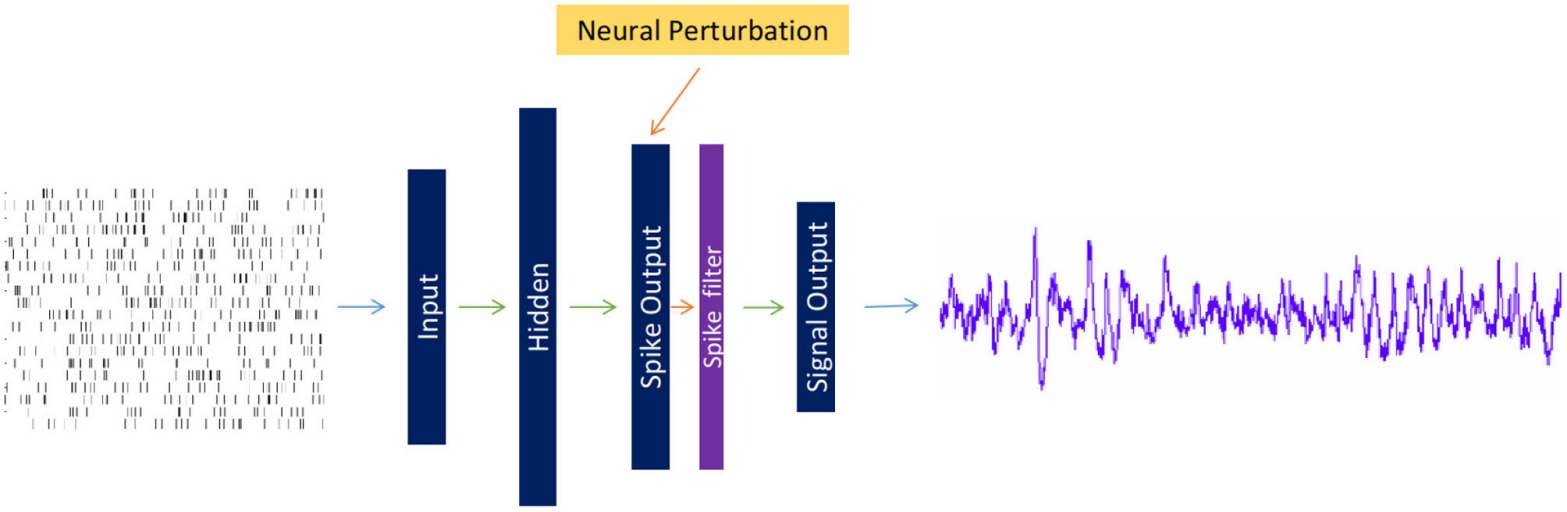
Depiction of a three-layered feedforward spiking network mapping a random spike train to an electroencephalography (EEG) signal with additional neural perturbation layer of Poisson neurons distorting the information of Spike Output layer. The spike train from the Spike Output layer is converted to a smoothed signal via a double exponential spike filter, which is then transformed to an EEG signal. The number of nodes in each layer varies depending on the task for best fit.

A feedforward spiking neural network, as depicted in Figure 1, with each node behaving as a Leaky-Integrate and Fire (LIF) neuron model, was designed for producing EEG signals. As the ground truth spike train triggering a targeted EEG signal is inaccessible, a Poisson-generated spike train with a firing rate of 10 Hz acts as the input layer preceding with one hidden and one output layer. The number of neurons in each layer is different for MI and SSVEP reconstruction (see Table 1). The spiking activity from the output layer is transformed to a rate signal using a double exponential synaptic filter (see Equation 9), which is then weighted averaged to produce an EEG signal. All the weight matrices between layers except for perturbation layers were trained during the reconstruction process. The number of nodes in the signal output layer varies depending on the number of EEG channels that were target toward generation. The perturbation layer containing Poisson neurons acts as background noise whose parameters i.e., the number of noise neurons and its firing rate, are varied to assess the effect of SNN on artificial data.

**Table 1:** List of parameters in the model equations, the spiking neural network (SNN) architecture, and the optimizer.

**Equation parameters**	**value**
*dt*	1 mS
*u*_*rest*_	0 mV
τ_*m*_	10 mS
τ_*s*_	5 mS
*R*	1 M
ϑ	1 mV
τ_*d*_	2 mS
τ_*r*_	3 mS
**Model parameters**	**Spiking layer nodes**
MI	[500, 1500, 100]
SSVEP	[100, 500, 200]
**Optimizer properties**	**Value**
Learning rate	0.0005
Betas	(0.9, 0.999)
Epsilon	8 *x* 10^−1^
Weight decay	0

*Spiking layer nodes represents an array of the number of neurons in each of the spiking network's layer*.

**Figure 2:**
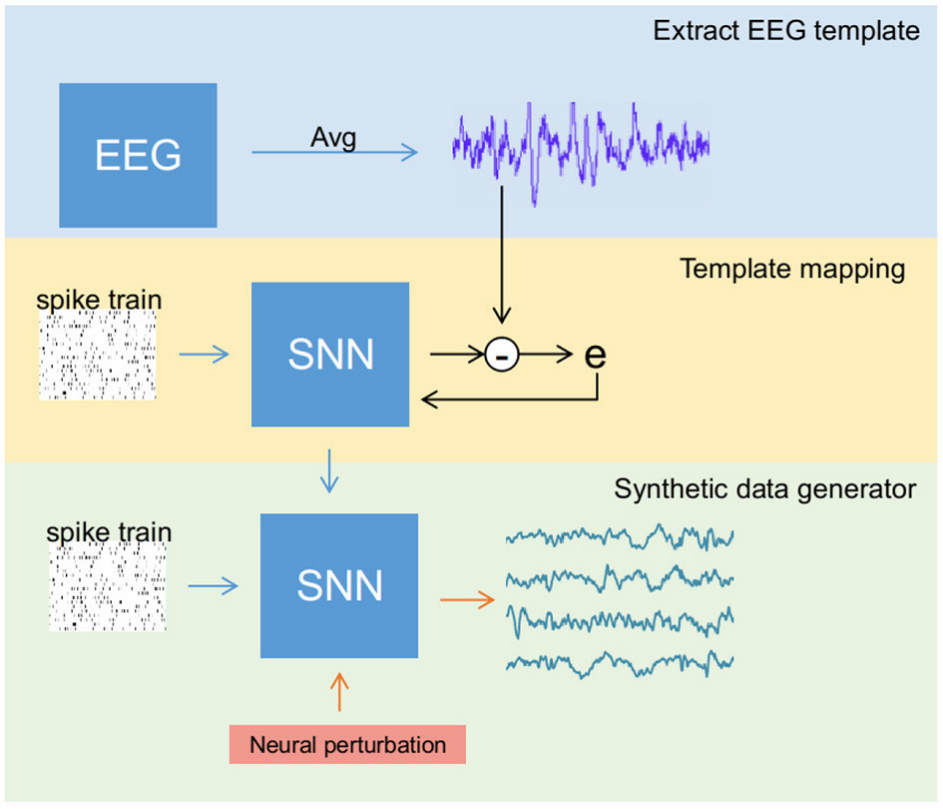
Overview of the steps involved in generating synthetic electroencephalography (EEG) samples using the spiking neural network (SNN). The process is divided into three different stages: (1) Template extraction, where EEG template from the sample data is derived (blue box), (2) training SNN to produce EEG signal template (yellow box), and (3) activating the perturbation layers that help SNN to generate artificial samples (green box).

The entire data generation process (see Figure 2) is performed in three stages: (a) extracting reliable EEG templates (one for each class) either through averaging original samples (in SSVEP) or selecting the best trial among all the samples (in MI), (b) training the SNN to reconstruct EEG templates of all classes simultaneously, and (c) activating the perturbation layer after completion of the training process to generate synthetic EEG samples. These stages were performed in the mentioned sequential order. In any recording, EEG signals for a given stimulus vary due to noise, artifacts, and background activity. However, under ideal conditions, all the trials retain the stimulus-related biomarker. Therefore, for stage 1 (i.e., template extraction), we averaged few trials to reduce noise and obtain a clean signal with a good biomarker (a strong power peak at target frequency in the power spectrum) in the case of SSVEP. Contrarily for MI, averaging impaired its biomarker alongside noise as the MI's biomarker spread in a range of mu and beta spectral bands. Therefore, we chose the best trial in a dataset as a template.

To extract a reliable EEG template in MI, a classification algorithm was applied on the original dataset for both training and testing. During testing, the probability of each sample to identify its class or label is estimated. The sample with highest probability (one for each class) was considered as template. For example, if two samples have probability of 0.9 and 0.8 to be labeled into a particular class, then the sample with highest probability (i.e., sample with 0.9 probability) is assumed as reliable template due to its high value.

Using the surrogate-gradient descent method for weight updating, the SNN is initially trained to reconstruct an EEG template. Then, the neural perturbation layer composed of Poisson neurons (acting as background activity) injects random current (a random spike train for each new sample) to the output layer via fixed synaptic weights, enabling the network to generate synthetic EEG samples that differ from the original signal while retaining its biomarker.

### 2.1. Neuron Model

Spiking neural networks are constructed with neurons that show biological realism. A wide range of neuron models exists with varying complexity, such as the Hodgkin Huxley (HH), the LIF neuron, the FitzHugh-Nagumo model, and the Izhikevich model. Opting for computational simplicity, the LIF neuron model, whose membrane potential is described by Equation (1), was chosen as a basis for constructing each node in a spiking layer.

(1) $$\begin{array}{r} \;\tau_{\text{m}}\frac{\text{d}u_{i}}{\text{d}t}=-\left(u_{i}-u_{\text{rest}}\right)+RI_{i} \end{array}$$

(2) $$\begin{array}{r} \frac{\text{d}I_{i}}{\text{d}t}=-\frac{I_{i}\left(t\right)}{\tau_{\text{s}}}+\underset{j}{\sum }W_{ij}S_{j}\left(t\right) \end{array}$$

where *u*_*i*_(*t*) is the membrane potential of a neuron, *i*, *u*_*rest*_ is the resting potential, τ_*m*_ is the membrane time constant, *R* is the input resistance, *I*_*i*_(*t*) is the input current, and τ_*s*_ is the synaptic time constant. The membrane potential increases with input current, and after reaching a threshold ϑ, the neuron emits a spike and resets its potential to *u*_*rest*_. Incorporating the reset property into Equation (1):

(3) $$\begin{array}{r} \;\frac{\text{d}u_{i}}{\text{d}t}=-\frac{1}{\tau_{\text{m}}}\left(u_{i}-u_{\text{rest}}\right)+RI_{i}+S_{i}\left(t\right)\left(u_{\text{rest}}-\vartheta \right) \end{array}$$

(4) $$\begin{array}{r} \;S_{i}\left(t\right)=\underset{k}{\sum }\delta \left(t-t_{i}^{k}\right) \end{array}$$

*S*_*i*_(*t*) is the spike train (sum of a dirac delta function, δ) of a neuron and $t_{i}^{k}$ is the *k*^*th*^ firing time of the corresponding neuron (see Equation 4). As suggested in Neftci et al. (2019), an approximated version of the LIF membrane potential for a small simulation time step Δ_*t*_ > 0, as shown in Equation (6), with the substitution of numerical value of *R*, *u*_*rest*_, and ϑ from Table 1, was implemented in this study.

(5) $$\begin{array}{r} I_{i}\left[n+1\right]=\alpha I_{i}\left[n\right]+\underset{j}{\sum }W_{ij}S_{j}\left[n\right] \end{array}$$

(6) $$\begin{array}{r} u_{i}\left[n+1\right]=\beta u_{i}\left[n\right]+I_{i}\left[n\right]-S_{i}\left[n\right] \end{array}$$

where $\alpha \equiv \exp\left(-\frac{\Delta_{t}}{\tau_{\text{s}}}\right)$, $\beta \equiv \exp\left(-\frac{\Delta_{t}}{\tau_{\text{m}}}\right)$.

Two other neuron models, the quadratic integrate-and-fire (QIF) model (Equation 7) and the exponential integrate-and-fire (EIF) model (Equation 8), were also tested for EEG reconstruction (see Supplementary Figures 2, 3).

(7) $$\begin{array}{r} \tau_{\text{m}}\frac{\text{d}}{\text{d}t}u_{i}=a_{0}\left(u_{i}-u_{\text{rest}}\right)\left(u_{i}-u_{c}\right)+RI_{i} \end{array}$$

(8) $$\begin{array}{r} \tau_{\text{m}}\frac{\text{d}}{\text{d}t}u_{i}=-\left(u_{i}-u_{\text{rest}}\right)+\Delta_{T}\exp\left(\frac{u_{i}-\vartheta }{\Delta_{T}}\right)+RI_{i} \end{array}$$

where, *a*_0_ > 0, *u*_*c*_ > *u*_rest_ and sharpness parameter Δ_*T*_ > 0. And, *I*_*i*_ follows the same dynamics as Equation (2).

### 2.2. Synaptic Filter

Spike trains of the output layer are filtered by a double exponential synaptic filter *r*_*j*_ for each neuron *j* as shown in the following equation:

(9) $$\begin{array}{l} \;{\dot{r}}_{j}=-\frac{r_{j}}{\tau_{d}}+h_{j}\\ {\dot{h}}_{j}=-\frac{h_{j}}{\tau_{r}}+\frac{1}{\tau_{r}\tau_{d}}\sum_{t_{j}<t}\delta \left(t-t_{jk}\right) \end{array}$$

where τ_*r*_ is the synaptic rise time and τ_*d*_ is the synaptic decay time. δ is the spike train of *Spike Output* layer (from Figure 1), which is filtered through an intermediary exponential filter *h*_*j*_ and in-turn via another exponential filter *r*_*j*_, which is the final output of the synaptic filter. Following the work of Nicola and Clopath (2017), in which a double exponential synaptic filter was applied to produce smoothed and continuous signals from spike trains, we opted to apply this filter to each output node in the final spiking layer of SNN. This filtered output is transformed into the EEG signal (single-channel EEG for SSVEP and two-channel EEG for MI) according, as shown in the following equation for a single channel *c*, where * indicates dot product multiplication.

(10) $$\begin{array}{r} EEG_{c}=\Sigma_{j}W_{cj}*r_{j} \end{array}$$

See Table 1 for a list of the model parameters.

### 2.3. Surrogate-Gradient Descent

A spike is emitted when the membrane voltage reaches a pre-defined threshold ϑ and reset back to resting potential and this behavior mimics Heaviside step activation function (Θ). Due to this non-differentiable nature of spiking neurons, training the synaptic weights is challenging as the traditional gradient descent algorithm commonly used for training artificial neural networks (ANNs) is unsuitable because the gradient is zero everywhere except at the event of spike emissions where it is undefined. Additionally, to account for the less extensive hyper-parameter search, in contrast to previous studies such as FORCE, SNNs in this study were trained using a surrogate-gradient descent approach (Zenke and Ganguli, 2018; Neftci et al., 2019), which introduces a continuous relaxation on gradient estimation without affecting the forward pass of spike trains. The step nature of spike emission *S*_*i*_[*n*] ∝ Θ(*u*_*i*_[*n*] − ϑ) from forward propagation is replaced with σ(*u*_*i*_[*n*] − ϑ) during backpropagation, where σ(*x*) = 1/(1 + *exp*(−*x*)). Finally, Adam optimizer (see Table 1 for properties) was adopted for weight optimization alongside the surrogate gradient.

### 2.4. EEG Datasets

For MI, the majority of the datasets is from publicly available BCI competition IV resources (Tangermann et al., 2012) and few datasets are previously recorded data by our group from Ko et al. (2019). All MI datasets are of 3 s trial duration, sampled to 250 Hz, and contain epochs of left-hand motor imagery and right-hand motor imagery classes. Similarly, publicly available datasets from Wang et al. (2016) were imported for SSVEP analysis. For SSVEP processing, only 2 s epochs were extracted and down-sampled to 250 Hz from the original datasets. Further EEG pre-processing steps for each modality are described in section 3.

### 2.5. Classification

Since SSVEP is a more effective and high performing BCI modality than motor imagery, this efficacy of data augmentation was explored toward MI classification assessment. Different perturbation combinations (number of noise neurons and their firing rate) in the perturbation layer were used to generate 100 samples (per combination) of artificial MI-EEG data. The artificial MI-EEG data are further assessed for classification performance by (1) comparing the cross-classification between artificial and original data, and (2) correlation analysis to compare the similarity of artificial data with the original. Common spatial pattern (CSP), a widely adopted method for extracting MI-related features (Wang et al., 2006) was used to test the above assessment and the details of the CSP algorithm is described in section 2.5.1. Finally, the features obtained via CSP were classified using linear discriminant analysis (LDA), support-vector machines (SVM) with linear kernel, K-nearest neighbor (K-NN) with k = 5, and Gaussian classifier were adopted (Mishuhina and Jiang, 2018), (Xygonakis et al., 2018).

#### 2.5.1. Common Spatial Pattern

The objective of CSP is to estimate spatial filters for maximal variance to discriminate two sets of data. With *E* as EEG signal and *W* as spatial filter, the transformed signal *S* is given by:

(11) $$\begin{array}{r} S=W^{T}E\;\text{or}\;s\left(t\right)=W^{T}e\left(t\right) \end{array}$$

The criteria for CSP is as follows:

(12) $$\begin{array}{l} \begin{array}{ll} \text{maximize} & \mathrm{tr}W^{T}\Sigma_{1}W\\ \text{subject to} & W^{T}\left(\Sigma_{1}+\Sigma_{2}\right)W=I \end{array} \end{array}$$

where

(13) $$\begin{array}{l} \begin{array}{l} \Sigma_{1}=\underset{E_{n}\in \left\{\text{class}1\right\}}{\mathrm{Exp}}\frac{E_{n}E_{n}^{T}}{\mathrm{tr}E_{n}E_{n}^{T}}\\ \Sigma_{2}=\underset{E_{n}\in \left\{\text{class}2\right\}}{\mathrm{Exp}}\frac{E_{n}E_{n}^{T}}{\mathrm{tr}E_{n}E_{n}^{T}} \end{array} \end{array}$$

## 3. Results

### 3.1. SNN for MI

Prominent MI features, such as event-related synchronization (ERS) and event-related desynchronization (ERD), are typically observed over the motor cortex and traced at central EEG electrodes (typically from C3 and C4 channels) in the mu frequency range of 8–13 Hz (Thomas et al., 2009; Shahid et al., 2010). Therefore, in this study, C3 and C4 channel time-series EEG data of two classes: (1) left-hand MI (LH-MI) and (2) right-hand MI (RH-MI) were targeted for data augmentation. The data are band-pass filtered between 0.5 and 20 Hz and sampled at 250 Hz in pre-processing. For extracting the target template, CSP was used to find the best sample from this processed data based on the probability of each sample to be classified accurately. As previously stated, a 10 Hz Poisson spike train matrix is provided as input, one fixed input for each class, and the SNN is trained using surrogate-gradient to produce the C3, C4 channel template. However, during this training process, the perturbation layer (in Figure 1) remains inactive.

**Figure 3:**
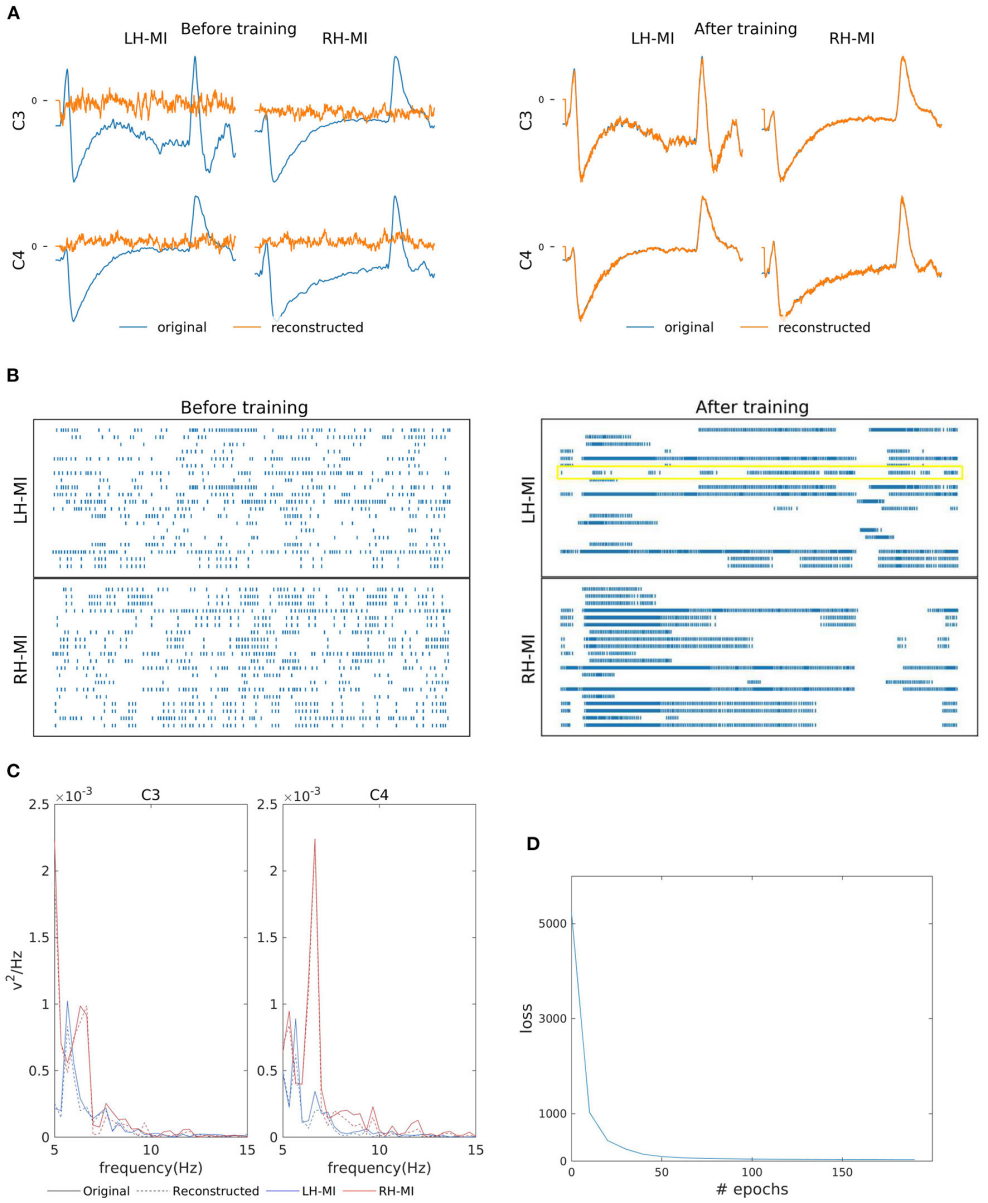
Left-Hand-MI (LH-MI) and Right-Hand MI (RH-MI) EEG signal reconstruction outcomes. **(A)** Illustration of original (blue line) and reconstructed (red line) C3 and C4 EEG signals for LH-MI and RH-MI tasks before and after SNN training. **(B)** Raster plot of 20 randomly selected neuronal spike trains from the hidden layer corresponding to both classes before and after training. The yellow box indicates the effectiveness of the training process in reorganizing the spike trains across layers. However, it should be noted that multiple neuronal spike trains rather than a single neuron contribute to the expected output signal. **(C)** Original (bold line) and reconstructed (dashed line) signal power spectrum at C3 and C4 channels for LH-MI (blue) and RH-MI (red), which appear to be fairly similar. **(D)** The loss value, i.e., the MSE, decreased substantially within a few epochs of the training process.

Figures 3A,B shows the two-channel EEG reconstruction from the training (template) signal before and after the training process and the corresponding reorganization neuron's spiking information from the hidden layer (see the yellow box in Figure 3B, indicating that the learning process is effective in reorganizing the spike trains after training). As previously mentioned, for MI reconstruction, C3 and C4 channel data generated by SNN (orange line in Figure 3A) were initially random and eventually converged to the EEG template (blue line). We further verified the similarity of reconstructed power spectra to that of the training signals as shown in Figure 3C, where the bold lines present the original power spectrum and dashed line represent the reconstructed line for each channel, i.e., C3 and C4 individually for LH-MI (blue line) and RH-MI (red line). Figure 3D shows that the loss, i.e., the mean squared error (MSE) between the SNN generated signal and the template signal, converges quickly after a few iterations or epochs. This trained model is later used for generating multiple artificial signals by activating the perturbation layer.

### 3.2. SNN for SSVEP

SSVEP is the other highly utilized modality for BCI communications. It is detected by the presence of contrasting high power at a frequency similar to the flickering stimulus visually focused on by a user. SSVEP is usually detected from the occipital region, and recordings are typically analyzed from the O1, O2, and Oz electrodes (Zhang et al., 2013). For example, if a user focuses on a 15 Hz flickering stimulus, the EEG signal from the occipital region displays high power at 15 Hz in the frequency domain. Since SSVEP has a high signal-to-noise ratio and is easily traceable with a single electrode, Oz channels data of 10 and 11 Hz SSVEP was considered for SNN-based reconstruction. Contrary to MI, the template signal from SSVEP data is extracted by averaging the available samples. Similar to MI template reconstruction, one fixed 10 Hz Poisson spike train matrix for each class is used as input for reconstructing 10 and 11 Hz Oz's SSVEP signal. Since the targeted frequency lies in the low-frequency range, the dataset was band-pass filtered between 0.5 and 20 Hz and has a sampling rate of 250 Hz.

**Figure 4:**
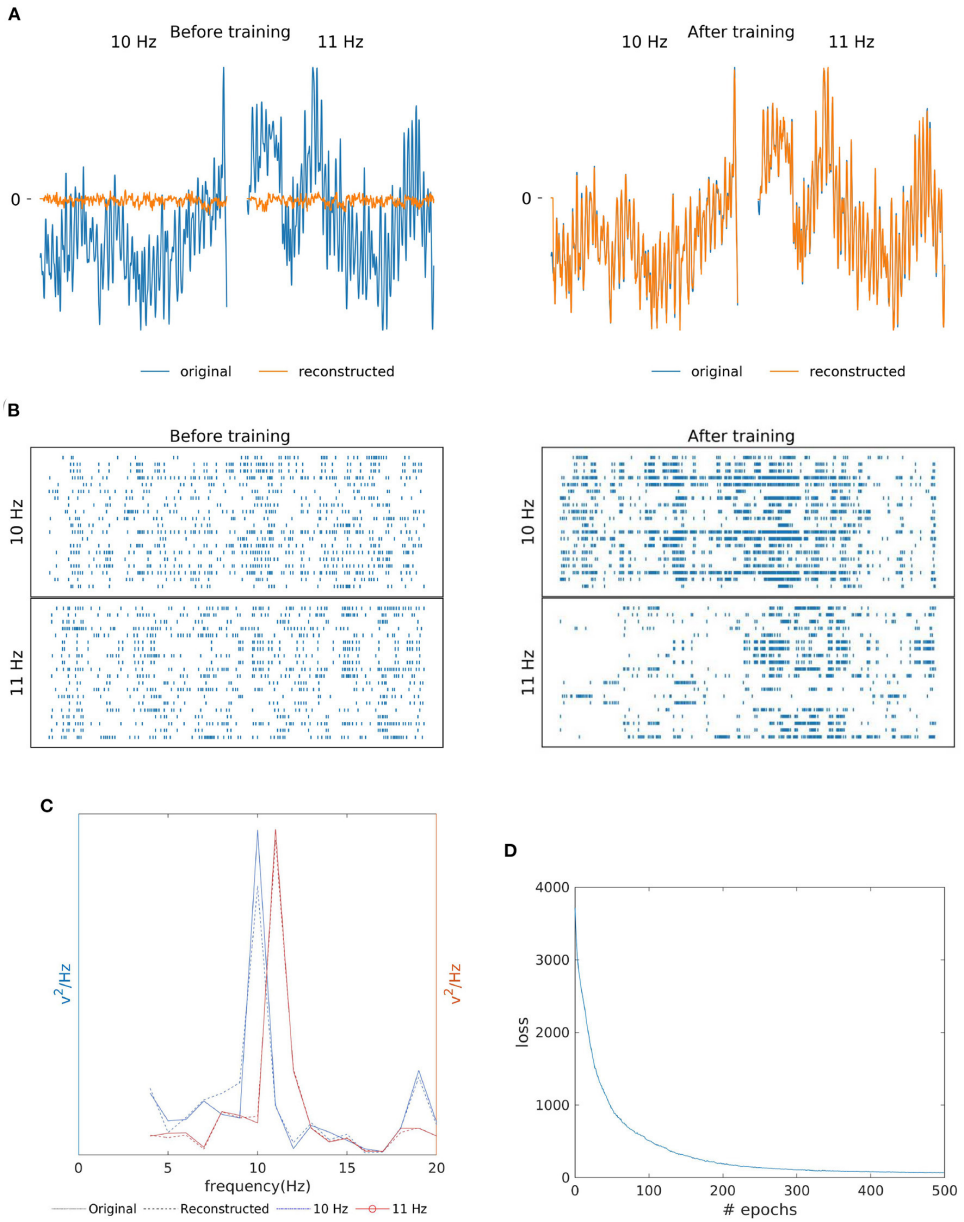
Two class SSVEP signal (10 and 11 Hz) reconstruction outcomes. **(A)** Illustration of original (blue line) and reconstructed (orange line) SSVEP signal of Oz channel before and after SNN training. **(B)** Raster plot of 20 randomly selected neuronal spike trains from the hidden layer corresponding to each class before and after training. The yellow box shows that the training process was effective at reorganizing spike trains. **(C)** Original (bold line) and reconstructed (dashed line) power spectra if signals recorded from the Oz channel are similar, indicating the preservation of the SSVEP biomarker in the reconstructed signal. **(D)** The loss value (MSE) across epochs during the training process.

As shown in Figures 4A,B, the similarity of the original and reconstructed signal and hidden layer's spike reorganization before and after SNN training. The SNN training processes were able to successfully re-produce EEG signal (orange line in Figure 4A) closely matching the original template (blue line). Additionally, Figure 4C shows the resemblance of the reconstructed signal's power spectrum (dashed line) to that of the training signal (bold line). And, for both the 10 Hz signal (blue line) and 11 Hz signal (red line), the reconstruction has the presence of a strong peak (an SSVEP biomarker) at their respective frequencies in the power spectrum. According to Figure 4D, similar to MI, the loss (MSE), converged within a few epochs of the training process.

### 3.3. Synthetic EEG Signal Validation

After successfully training the SNN to reconstruct EEG signal, the neural perturbation layer was activated to generate synthetic EEG data. The number of Poisson neurons (or noise neurons) and the firing rate (or noise frequency) were varied to generate synthetic data in different variance levels. To validate the synthetic data, 100 samples per class were generated for a given combination of neurons and their firing frequency.

**Figure 5:**
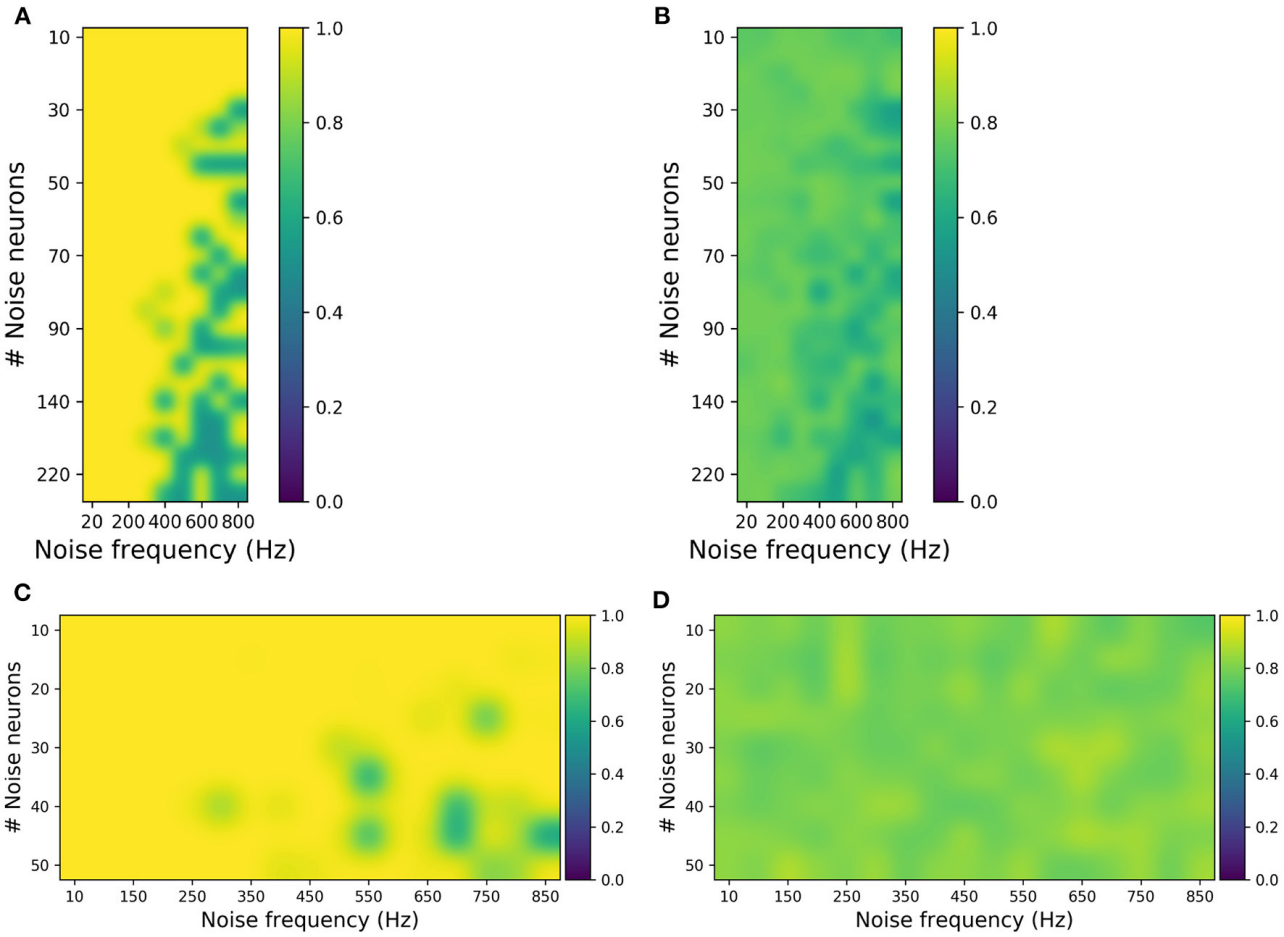
Evaluating synthetic motor imagery (MI) and steady-state visually evoked potential (SSVEP) data generated from different combinations of the number of noise neurons and the firing frequency in the perturbation layer. **(A,B)** MI accuracy obtained by training the classifier with original data and testing with synthetic data (**A**) and vice versa (**B**). **(C,D)** SSVEP accuracy obtained by training classifier with original data and testing with synthetic data **(C)** and vice versa **(D)**. The highest accuracy in **(B)** is ~80%, while the highest accuracy in **(D)** is 90%, which are close to the accuracies obtained when the original data are used for both training and testing the classifier.

Two validation procedures were implemented, *validation-a*: a classifier was trained with the original dataset and tested with the synthetic dataset, and *validation-b*: vice-versa, i.e., a classifier is trained with synthetic data and tested with original data. This validation was performed independently with the data generated by each perturbation combination (see Figure 5). The classifiers used for validating artificial MI and SSVEP data include CSP with LDA and multi-layer perceptron (MLP), respectively.

For MI, from Figure 5A, it was observed that the synthetic EEG can be classified accurately when the classifier is trained with original data (validation-a). Figure 5B shows that although the classification accuracies are not high (for validation-b), the highest accuracy of approximately 80% is similar to the performance when the original dataset is used both for training and testing. And these validations were done independently for each perturbation combination, ranging from 20 to 800 Hz noise frequency and 10–220 noise neurons. This suggests that the generated synthetic data covers the broad variance of the EEG samples. Similarly for SSVEP, Figures 5C,D has shown that the synthetic SSVEP passed the validation check with high accuracy. In addition to successful classification, the synthetic data yielded the highest accuracy of approximately 90% when used as a training dataset.

For SSVEP, the perturbation covers a range of 10–50 noise neurons and 10–850 Hz noise frequency. And, similar to MI, SSVEP synthetic validation was done independently for each perturbation combination. Overall, these validation results show that, in addition to reconstructing the EEG signal, the SNN can be used to generate synthetic EEG samples that are in agreement with the original samples.

**Figure 6:**
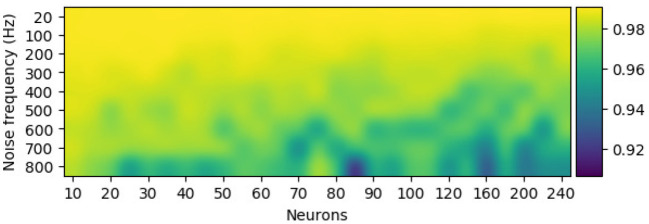
The configuration of the perturbation layer, i.e., the number of neurons and their firing rate, modulates the similarity of the synthetically generated sample to its template for the C4 electroencephalography (EEG) signal. Pearson's correlation between the averaged synthetic samples (100 samples) and the template showcase higher deviation from the template with more neurons and a high firing rate in the perturbation layer.

The configuration of the perturbation layer (i.e., the combination of the number of noise neurons and its firing rate) can be changed to generate data at various scales (or different levels of noise) from the template. Few neurons with low firing rates tend to produce EEG trials that are highly similar to the template, while a perturbation layer with a huge number of neurons and a high firing rate completely distort the synthetic samples of the template. To further check the effect of perturbation combination of artificial data, Pearson's correlation, a metric for estimating the linear correlation between two variables, has been implemented. Figure 6 shows that for a given EEG channel of a given class (in this case, RH-MI's C4 channel), the correlation between the averaged (100 samples) synthetic sample and the template is inversely proportional to the number of neurons and the firing rate of the perturbation layer.

### 3.4. Classification

Since detecting reliable MI features is difficult in comparison to detecting SSVEP, we focused on the classification efficacy of MI-BCI using artificial data. Therefore, for assessing synthetic MI-EEG data, similar to validation-b, a classifier was trained with synthetic data (100 samples per class) and tested with original data. For a baseline comparison, a 100-fold cross-validation process was applied for the original data, in which original data were used for both training and testing. For each fold, 95% of the original data (samples are randomly selected) form a training set and the remaining 5% of the original data were labeled as a test set. This process is repeated for each fold and average accuracy across the fold is estimated to be the baseline accuracy. The synthetic data generated by the perturbation configuration that resulted in the highest accuracy during the validation process (from Figure 5B) was used as the final artificial dataset. This process is verified with multiple publicly available datasets (see section 2.4).

Table 2 showcases the accuracy obtained by synthetic data with LDA, SVM, KNN, and Gaussian classifiers. Each classifier was implemented on a dataset basis and in total 13 different datasets were used in this study. When trained with synthetic data, all the classifier was able to successfully predict the labels of original samples and the overall performance was comparable to baseline performance. In particular, LDA resulted in the highest baseline performance (of 70.78%) as commonly stated in literature and the generated synthetic data enhanced the performance to 72.71% with LDA. Considering all the classifiers, a given dataset has shown enhanced performance with synthetic data. Interestingly, for few datasets, the synthetic dataset able to enhance the classification performance up to 9% (for the example dataset 4 with Gaussian classifier). As a cross-validation approach was used to obtain each dataset's baseline accuracy and the distribution of these accuracies across the datasets is unknown, a Wilcoxon signed-rank test was implemented for statistical significance analysis (between performance with synthetic data and original data) and obtained a *p*-value < 0.01 for all the classifiers.

**Table 2:** Comparison of classification performance for synthetic motor imagery (MI) assessment when (a) the classifier is trained and tested with original data using 100-fold cross-validation (95% training set and 5% testing set) for baseline accuracy estimation (denoted as *Original*), and (b) the classifier is trained with synthetic data and tested with all of the original data (denoted as *Synthetic*).

**Dataset**	**LDA**		**SVM**		**KNN**		**Gaussian**	
	**Original**	**Synthetic**	**Original**	**Synthetic**	**Original**	**Synthetic**	**Original**	**Synthetic**
1	80.45	82.50	79.00	**83.13**	81.89	**83.13**	78.44	81.25
2	59.11	63.13	61.56	63.13	54.67	63.13	61.11	**63.75**
3	52.22	**61.25**	51.78	60.63	43.44	60.63	56.56	60.00
4	78.38	67.36	77.63	79.17	74.50	**81.94**	70.13	79.17
5	60.00	65.28	63.00	65.97	**67.75**	66.67	52.75	58.33
6	64.75	65.63	61.88	65.63	56.13	64.38	64.50	**68.75**
7	60.05	**65.83**	62.83	65.00	65.33	63.33	51.83	65.00
8	79.88	**80.71**	78.13	80.71	69.63	80.00	54.00	**80.71**
9	91.00	**93.13**	91.38	93.13	91.00	**93.13**	90.63	91.25
10	71.63	70.63	70.63	69.38	71.50	**72.50**	71.13	**72.50**
11	82.88	**85.00**	83.25	84.38	83.00	84.38	81.00	84.38
12	78.17	77.50	76.17	**78.33**	70.17	**78.33**	69.00	77.50
13	61.63	67.36	59.38	66.67	62.50	67.36	60.88	**70.14**
**Avg**	70.78	**72.71**	70.51	**73.48**	68.58	**73.76**	66.30	**73.29**

*Common spatial pattern (CSP) was used to extract features and was classified with linear discriminant analysis (LDA), support-vector machines (SVM), K-nearest neighbor (KNN), and Gaussian classifier. For all the classifiers, Synthetic has resulted in enhanced performance. LDA resulted in the highest baseline performance of 70.78% and the performance was improved to 72.71% with Synthetic. A Wilcoxon signed-rank test resulted in p-value < 0.01 for all the classifiers. Bold represents the maximum value obtained by a dataset*.

## 4. Discussion

Understanding how chaotic neural activity reorganizes to allow humans to perform a wide range of activities, such as walking, running, and speech remains a topic of interest among the neuroscience community. Previous studies have trained models to produce such behavior using different learning methods, such as FORCE, both in artificial neural networks and spiking networks (Sussillo and Abbott, 2009; Nicola and Clopath, 2017; DePasquale et al., 2018). These enforced behaviors are periodic in nature, and training using non-stationary signals such as EEG, is challenging, especially if the algorithm requires a large hyper-parameter search space. Also, EEG signals vary for the same stimulus, i.e., no two EEG samples produced by the human brain are the same, although they retain their prominent biomarker (e.g., ERD/ERS rhythm for MI and high power at flickering stimulus frequency in SSVEP). Since EEG represents an accumulation of potentials of a spiking neuron, a primary focus of this study is to understand how the spiking neural network's activity is organized to form EEG signals and, in turn, generate different samples while retaining the necessary biomarker. Therefore, this study developed a procedure with feedforward SNN and surrogate gradient descent to reconstruct the EEG template in a supervised fashion and used neural perturbation to generate synthetic EEG signals. The proposed model was successfully applied for MI and SSVEP reconstruction, artificial data generation, and validated the synthetic EEG through classification metrics. Additionally, we showed that this network can be used with other BCI modalities, such as P300 (see Supplementary Figure 1), and is compatible with other neuron models at a computational cost (see Supplementary Figures 2, 3). To the best of our knowledge, this is the first study to generate synthetic EEG using SNN.

As previously stated, the primary aim of generating synthetic data from a machine learning perspective is to better train a classifier. As shown in Table 2, synthetic data can be utilized to enhance BCI performance, especially when the baseline performance of the dataset is low. Although the performance increment was not consistent among all datasets, the proposed approach yielded a significant increase in performance for certain datasets.

In the field of machine learning, the current state-of-the-art model for data augmentation is GAN and its variants. However, to generate artificial data, GAN requires a huge amount of training samples which we find to be counterintuitive. Also, existing EEG based GAN studies normalize the signal from 0~1 or −1~1 and often fail with EEG data at their original scales. In contrast, the proposed method needs only a few (one or two) samples for generating artificial data and can handle both normalized and non-normalized EEG signals. To this end, we compared recently published generative models and GAN variants, such as VAE, W-GAN, and DCGAN, and class-conditioned W-GAN (cc-WGAN) (Aznan et al., 2019; Panwar et al., 2020), with SNN for MI data generation and validation tests. The cc-WGAN model was used to generate MI data for few datasets. And previous studies usually mix the samples of original and artificial for classification enhancement often fail to perform *validation-b*. Appropriate synthetic data apart from enhancing the classification performance should also pass this validation check (see section 3.3). According to Supplementary Table 1, we observed that the GAN-generated data failed in this validation check with below chance level. The reason for this could be of multitude such as the random noise in the GAN-generated data improved the performance by chance, or limited amount of data (the GAN was trained independently for each dataset containing approximately 180 trials) failed to capture MI's biomarker. Contrarily, the proposed SNN approach passed this validation check (Supplementary Table 1) and delivered close to baseline or improved performance. Another major reason for this could be because, since SNN generates data based on a template signal, the artificial trials with varying distribution retrained MI-biomarker, and it in-turn able to make the classifier extract better features. Since GAN accounts for the whole dataset for training, the artifacts and noise present in the data could have lead to the generation of improper samples. This hypothesis could be explored in the future to fine-tune GAN's and SNN's training samples.

In agreement with previous studies, we interestingly observed a reorganization of neural activity (i.e., spike train) concerning the output signal. For example, the SSVEP signal at a defined frequency is slightly oscillatory (like a sine or cosine wave), which is reflected in the periodic spiking activity of certain neurons in the output layer (see Figure 4B). We further tested the proposed model for robustness by randomly clipping synapses and found that the overall signal trend was maintained despite the 30% reduction in synapses (see Supplementary Figure 4). According to Dale's law, a neuron is only either excitatory or inhibitory (Capano et al., 2015). However, this constraint was omitted from this study for simplicity, although it would be ideal to enforce the non-stationary behavior, such as in EEG, to spiking neural networks to maintain Dale's law in the future. Additionally, the current study, being focused on MI and SSVEP data, considered only a few channels. However, other minor BCI-related concepts such as fatigue, attention, and other physiological factors distribute their feature across a range of EEG channels. Therefore, another future direction of this study could be to test the limits of the model in terms of the number of channels and categories.

## Data Availability Statement

Publicly available datasets were analyzed in this study. This data can be found at: http://www.bbci.de/competition/iv/#dataset2a; http://www.bbci.de/competition/iv/#dataset2b; http://bci.med.tsinghua.edu.cn/download.html.

## Author Contributions

SS and C-TL conceptualized the concept and wrote the manuscript. SS wrote the code and performed the simulations. All authors contributed to the article and approved the submitted version.

## Conflict of Interest

The authors declare that the research was conducted in the absence of any commercial or financial relationships that could be construed as a potential conflict of interest.
