# Supplementary material for: Spiking Neural Network for Augmenting Electroencephalographic Data for Brain Computer Interfaces
Source: Front Neurosci. 2021 Apr 1;15:651762. doi: 10.3389/fnins.2021.651762 (PMC8047134; doi:10.3389/fnins.2021.651762)
Supplement: Supplementary file 1 [file Data_Sheet_1.PDF]

Supplementary Material

1 EEG RECONSTRUCTION

Apart from Motor Imagery (MI) and Steady State Visually Evoked Potential (SSVEP) signal, the proposed SNN model is verified to reconstruct other prominent BCI modality, P300. In addition, P300 was chosen to assess if SNN can handle sudden fluctuation (peak) in the target signal.

1.1 P300 signal reconstruction

From the figure below, it can be observed that the SNN can account for sudden and huge fluctuations in Event-Related Potentials (ERP) such as P300 whilst also learning non-ERP signals.

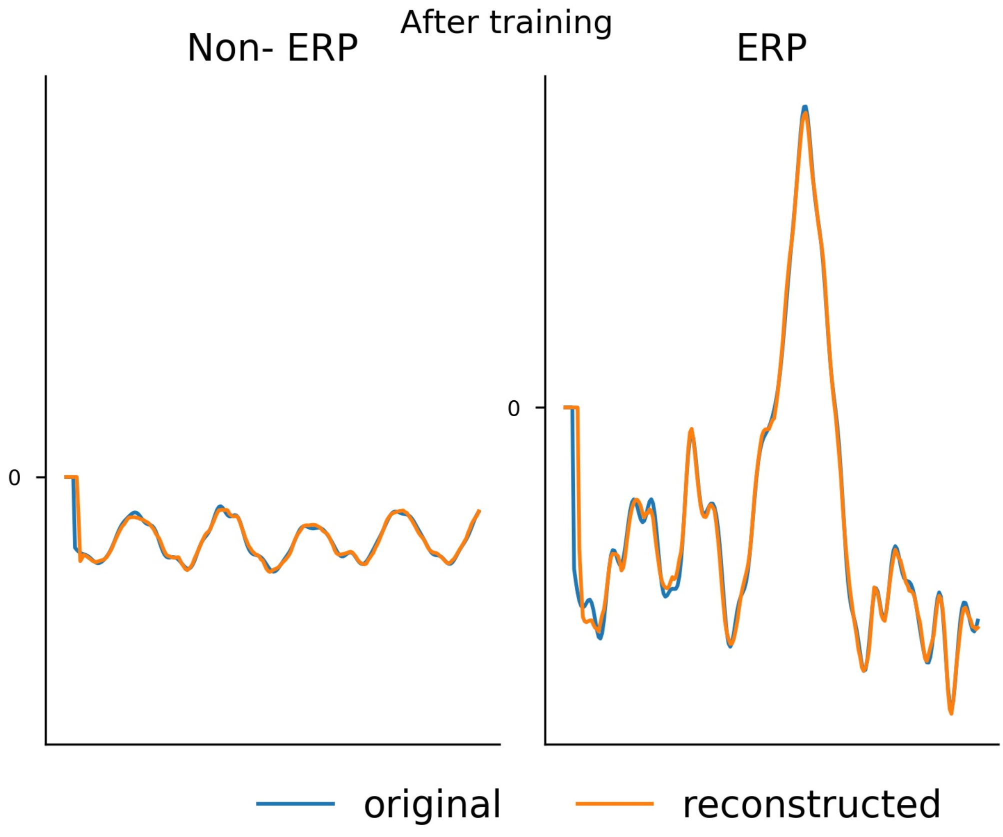

Figure S1. Reconstruction of P300 signal (orange line) using spiking neural network for Non-ERP and ERP signal compared to original template (blue line).

2 NEURON MODELS FOR SNN

LIF neuron model is the simplest and commonly used for constructing SNNs. In the field of computational neuroscience, other models such as QIF and EIF neuron model to understand the dynamics of neural circuitry. Here, we adopted these model to assess its ability for EEG signal reconstruction. To maintain simplicity, these neuron models were only verified to produce single channel (Oz) multi-class (12Hz and 15Hz) SSVEP signal as below.

(A)

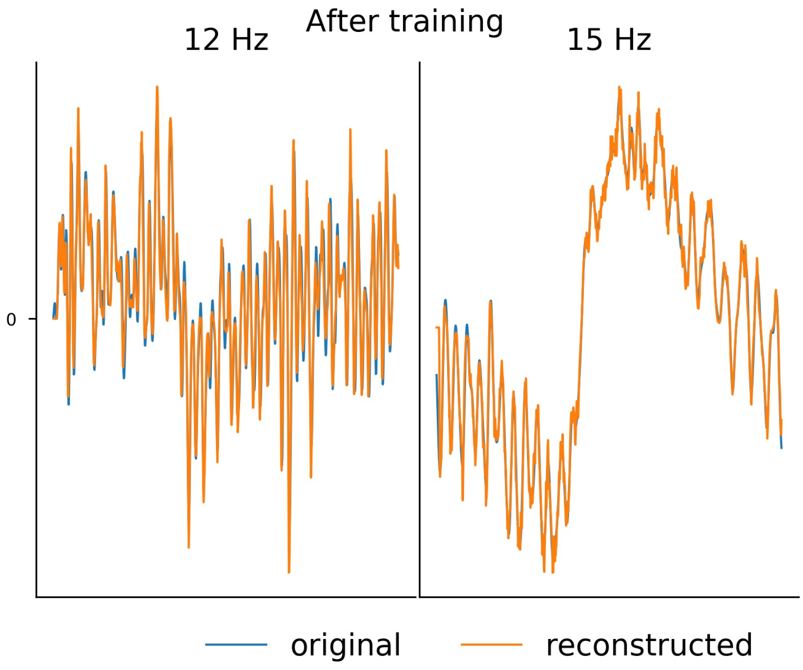

(B)

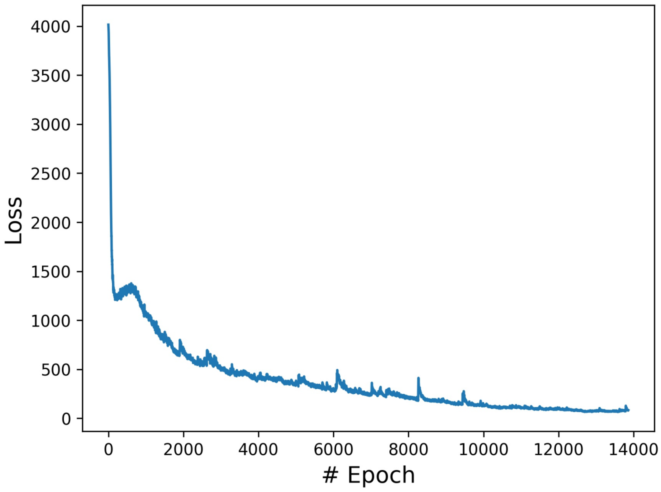

Figure S2. **(A)** Reconstruction (orange line) of 12 Hz and 15 Hz SSVEP EEG signal using QIF neuron model-based spiking neural network based on original template signal (blue line). **(B)** The decrease in loss during the training period.

(A)

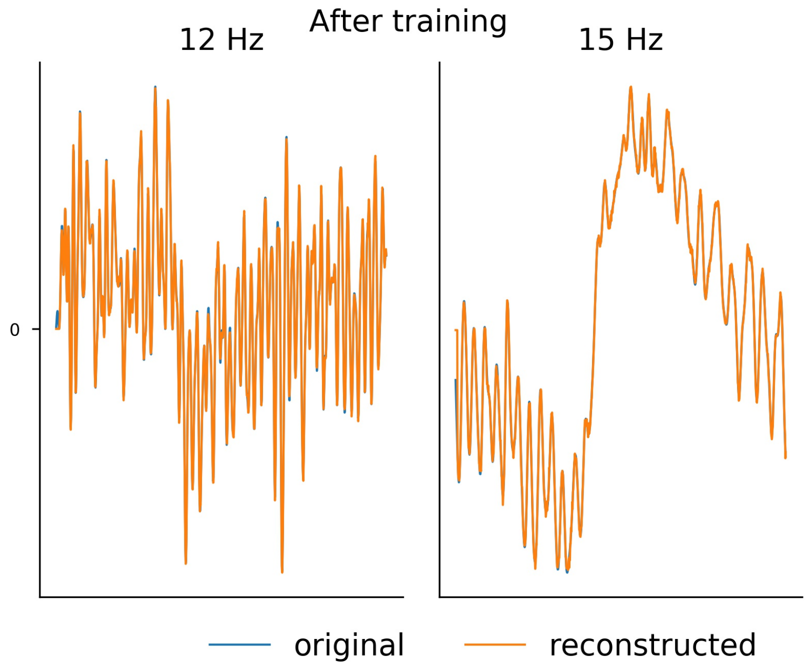

(B)

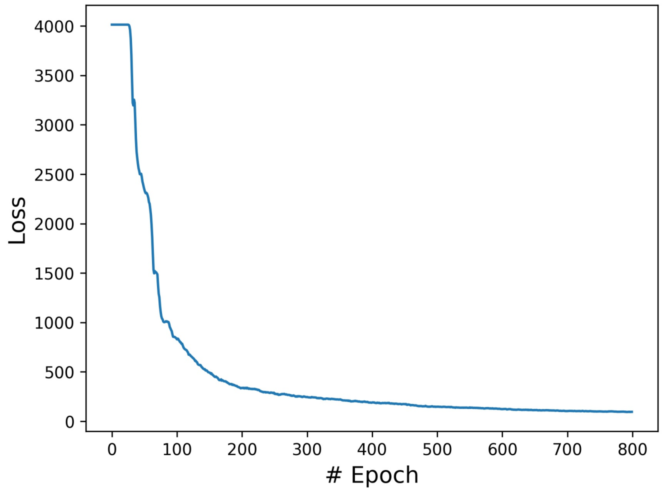

Figure S3. **(A)** Reconstruction (orange line) of 12 Hz and 15 Hz SSVEP EEG signal using EIF neuron model-based spiking neural network based on original template signal (blue line). **(B)** The decrease in loss during the training period.

3 ROBUSTNESS OF SNN

After SNN training to reconstruct a template EEG signal, the robustness of SNN model was tested by randomly disconnecting different fractions (from 10∼30 %) of the synapses of the hidden layer, i.e, randomly setting weight value to 0. 100 samples for each fraction for were recorded and averaged to observe the the similarity of final output signal to the template. As shown in below figure, the overall pattern of the time series signal remained in intact despite huge reduction in synapses.

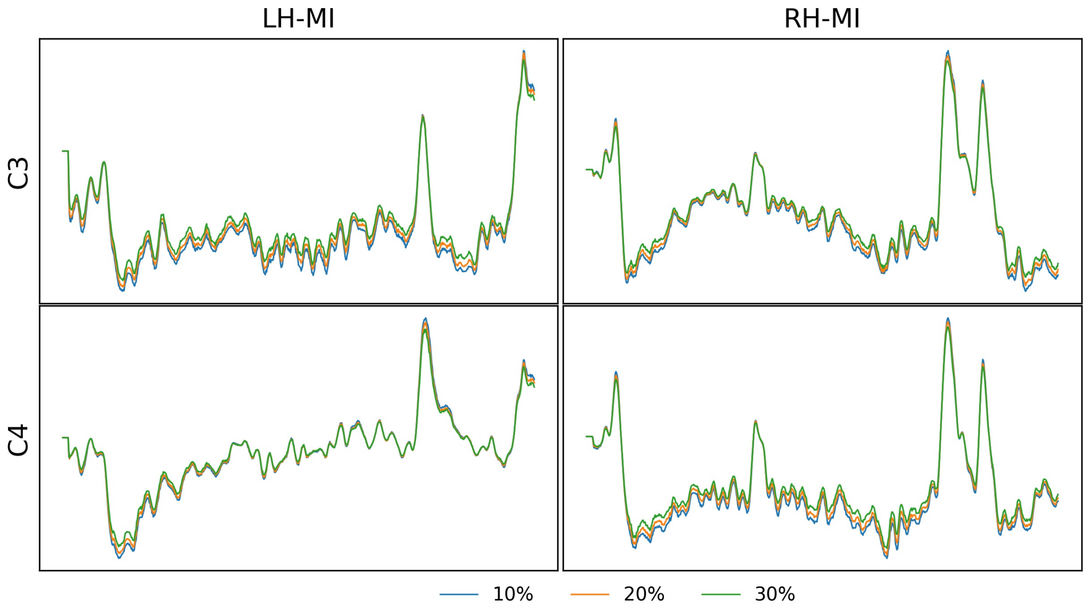

Figure S4. Robustness of the SNN model in MI reconstruction. Each sub plot represents the averaged output signal (100 samples) of a given channel (C3 or C4) for either RH-MI or LH-MI class produced by SNN. Each sample was produced by randomly disconnecting 10-30% of synapses (i.e., setting weight to 0).

4 COMPARING SNN AND GAN

GAN based EEG synthesis usually normalize EEG data from 0∼1 or -1 ∼ 1 and the proposed SNN model can function at the original scales of the recorded data. Past studies often ignore a validation check (see *validation-b* from section 3.3) and mix samples from original and artificial data in classification assessment. Any artificial data should pass this validation check to confirm an artificial data representing the targeted data. To this end, for fair comparison, data without normalization from different generative models such as VAE, DCGAN,W-GAN, and cc-GAN was considered for this check. From Table S1 below, it can be observed that GAN variants generated data's performance was below chance level and SNN generated data successfully passed this check.

Table S1. Validation of synthetic EEG signal generated by the proposed SNN method, VAE, DCGAN, W-GAN, and cc-WGAN. For this validation, the CSP with LDA was trained with synthetic data and tested with original data.

Dataset	VAE	DCGAN	W-GAN	cc-GAN	SNN
1	50.00	43.75	54.17	50.00	67.36
2	50.00	45.83	50.00	50.00	65.28
3	50.00	50.00	49.17	59.17	65.83
4	65.71	48.57	50.00	76.40	80.71
5	91.25	88.75	50.00	50.00	93.13
6	51.25	65.00	46.88	50.00	70.63
7	83.13	83.13	83.13	82.50	85.00
8	50.00	55.56	50.00	40.00	67.36
**Avg**	61.42	60.07	54.17	57.26	74.41

5 PSEUDOCODES

As mentioned previously, the entire proposed approach follows three step: template extraction, SNN training and data generation. For ease of readability pseudocode for each step is provided independently as below. Also, the python source code is provided in our github repository https://github.com/S-Kalyan/SNN_EEG.

Algorithm 1 Template Extraction

```
C: a classifier
D: a set containing EEG samples S_i
Train C with dataset D
procedure FOR ONE CLASS (j) OF D
    S^j ← list of samples of class j from D
    for each sample i in S^j do
        P_i^j = Estimate the probability of sample S_i^j belonging to class j
    end for
    K = arg max_i P_i^j
    Template T^j = S_K^j
end procedure
```

Algorithm 2 SNN Training with Template

```
Obtain EEG template T^j for each class j from Algorithm 1
I: initialize Input Spike Train
W: initialize weights in SNN
N: define number of epochs
lr: define learning rate
epoch: epoch number set to 0
while epoch is less than N do
    Estimate output spike train (S) from SNN: S = SNN(I)
    Obtain filtered spike train (r) from S using Equation 9
    Estimate predicted output EEG using Equation 10.
    L = mean square loss between EEG and T^j ∀j
    procedure IMPLEMENT SURROGATE-GRADIENT DESCENT
        for each spike estimation in S: replace Θ with σ
        Update weights: W ← W − lr ∗ ∇L
    end procedure
    epoch = epoch + 1
end while
```

Algorithm 3 SNN data generation

```
activate perturbation layer
nn: a list of number of noise neurons
nf: a list of firing rate of noise neurons
SNN: import trained SNN model from Algorithm 2
I: use Input Spike Train from Algorithm 2
for each element in nn do
    W: initialize random weight for perturbation layer
    for for each element in nf do
        procedure REPEAT FOR N TIMES FOR N ARTIFICIAL SAMPLES
            generate Poisson noise spike train (P) for the layer
            generate artificial EEG sample: SNN(I, W * P)
        end procedure
    end for
end for
```
